# CSINet: A Cross-Scale Interaction Network for Lightweight Image Super-Resolution

**DOI:** 10.3390/s24041135

**Published:** 2024-02-09

**Authors:** Gang Ke, Sio-Long Lo, Hua Zou, Yi-Feng Liu, Zhen-Qiang Chen, Jing-Kai Wang

**Affiliations:** 1 School of Computer Science and Engineering, Macau University of Science and Technology, Macau 999078, China; kegang95@126.com (G.K.); douglaslau@foxmail.com (Y.-F.L.); 3220002509@student.must.edu.mo (Z.-Q.C.); ming359046415@gmail.com (J.-K.W.); 2 School of Electronic Information, Dongguan Polytechnic, Dongguan 523109, China; 3 School of Computer Science, Wuhan University, Wuhan 430072, China; zouhua@whu.edu.cn

**Keywords:** super-resolution, cross-scale interaction, factorized convolution, efficient large convolutional kernel attention

## Abstract

In recent years, advancements in deep Convolutional Neural Networks (CNNs) have brought about a paradigm shift in the realm of image super-resolution (SR). While augmenting the depth and breadth of CNNs can indeed enhance network performance, it often comes at the expense of heightened computational demands and greater memory usage, which can restrict practical deployment. To mitigate this challenge, we have incorporated a technique called factorized convolution and introduced the efficient Cross-Scale Interaction Block (CSIB). CSIB employs a dual-branch structure, with one branch extracting local features and the other capturing global features. Interaction operations take place in the middle of this dual-branch structure, facilitating the integration of cross-scale contextual information. To further refine the aggregated contextual information, we designed an Efficient Large Kernel Attention (ELKA) using large convolutional kernels and a gating mechanism. By stacking CSIBs, we have created a lightweight cross-scale interaction network for image super-resolution named “CSINet”. This innovative approach significantly reduces computational costs while maintaining performance, providing an efficient solution for practical applications. The experimental results convincingly demonstrate that our CSINet surpasses the majority of the state-of-the-art lightweight super-resolution techniques used on widely recognized benchmark datasets. Moreover, our smaller model, CSINet-S, shows an excellent performance record on lightweight super-resolution benchmarks with extremely low parameters and Multi-Adds (e.g., 33.82 dB@Set14 × 2 with only 248 K parameters).

## 1. Introduction

Single image super resolution (SR) is a low-level task in the field of computer vision that aims to reconstruct a high resolution from a corresponding low-resolution image (LR), which is widely used in many applications, such as mobile devices, surveillance systems, autonomous driving, medical imaging, etc. However, SR is an ill-posed problem, since an identical LR image may be degenerated from different HR images. Therefore, SR is still a challenging task in terms of how to efficiently visually reconstruct HR images from degraded LR images.

To address this issue, Dong et al. [1] proposed SRCNN, marking the first application of deep learning methods in the field of single image super-resolution (SR). Achieving significantly superior results compared to traditional methods, SRCNN utilizes a three-layer convolutional neural network. To mitigate the computational demands of SRCNN, Kim et al. [2] introduced the VDSR model, incorporating residual learning to deepen the network to 20 layers and achieve rapid convergence. Lim et al. [3] presented the EDSR model, simplifying the network structure by removing batch normalization layers (BN), enhancing the model’s representational capacity, and securing victory in the NTIRE2017 Super-Resolution Challenge. Zhang et al. [4] proposed a residual-in-residual structure, pushing the depth of the Convolutional Neural Network (CNN) to 100–400 layers, yielding remarkably high PSNR values on benchmark datasets, surpassing previous methods.

While increasing the depth and width of CNNs can significantly enhance the network performance, it also comes with a significant computational cost and memory overhead, which restricts its use in practical applications like mobile devices, robots, edge computing, etc. In order to solve the issue, it is essential to develop a network that is both lightweight and incredibly effective.

Several prior methods [5,6,7,8,9,10] have been proposed in order to achieve a better trade-off between SR performance and computational efficiency. However, there are issues such as a small receptive field, slow convergence speed, information loss, and the fact that all network structures are constructed by a single-scale convolution kernel.

Recent research indicates that efficient operator design is crucial for building efficient SR CNNs. Factorized convolution, as an efficient operator, can break down standard convolution operations into two smaller convolution operations, effectively reducing the computational complexity and the number of parameters while maintaining network performance. However, there has been no prior study on efficient SR algorithms based on factorized convolution. Therefore, in this paper, we adopt a factorized convolution approach and construct an efficient Cross-Scale Interaction Block (CSIB).

The design rationale of CSIB is as follows: Firstly, we employ a 1×1 convolution layer to decrease the number of parameters and expedite the training process, followed by splitting them into two separate branches. To alleviate the computational burden, we employ factorized convolution to factorize the standard 5×5 depth-wise convolution into 5×1 and 1×5 depth-wise convolutions. One branch makes use of the factorized depth-wise convolution to extract local fine-grained features, while the other branch utilizes factorized depth-wise dilated convolution to capture global coarse-grained features. To prevent grid artifacts, different CSIB modules employ varying dilation rates. It is important to note that, for the more effective integration of cross-scale contextual information, we conduct interaction operations at the end of the dual-branch structure. This design not only reduces the computational complexity but also adeptly merges the local details and global features in the image. To further refine the aggregated contextual information, we designed an Efficient Large Convolutional Kernel Attention (ELKA) using large convolutional kernels and a gating mechanism.

**Figure 1:**
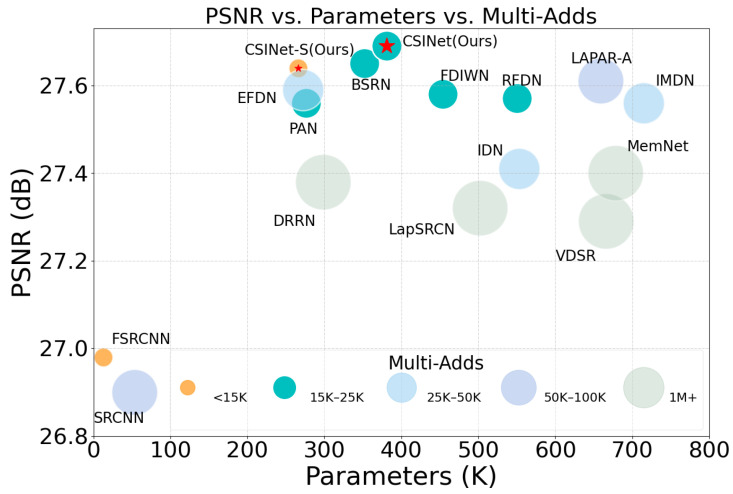
Trade-off between performance and model complexity of other state-of-the-art lightweight models on the BSD100 dataset for ×4 SR. The CSINet achieves higher PSNR with fewer parameters.

We built a Cross-Scale Interaction Network, named “CSINet”, by stacking CSIBs to extract multi-scale contextual information. This design balances the performance and computational efficiency, and our CSINet outperforms most lightweight SISR methods in terms of both the performance and computational complexity. Specific experimental results can be seen in Figure 1. This is a summary of the key contributions of our work:

1. We adopted a factorized convolution approach to design a Cross-Scale Interaction Block (CSIB). CSIBs employ a dual-branch structure to extract both local fine-grained features and global coarse-grained features. Furthermore, we utilize interaction operations at the end of the dual-branch structure, facilitating the integration of cross-scale contextual information;
2. We designed an Efficient Large Convolutional Kernel Attention (ELKA) with limited additional computation for refining and extracting features. Ablation studies validated the effectiveness of this attention module;
3. Comprehensive experiments on benchmark datasets show that our CSINet outperforms most state-of-the art lightweight SR methods.

## 2. Related Work

### 2.1. Lightweight Image SR

To improve the network speed while maintaining superior reconstruction results, several lightweight image super-resolution networks have been introduced [1,7,11,12,13,14]. These networks can be broadly categorized into three groups: network structure design, knowledge distillation, and pruning. In the network structure design methods, FSRCNN [1] is the first lightweight super-resolution model. It performs upsampling at the end of the network, significantly improving the processing speed, but the performance of image reconstruction still needs improvement. CARN [14] designs a cascaded residual module based on grouped convolution and adopts a mechanism of local and global cascading to fuse multi-layer features, thereby accelerating the model’s running speed. PAN [8] designs self-calibrated blocks with pixel attention and upsampling blocks, achieving competitive performance with only 272K parameters.

In knowledge distillation methods, IDN [15] uses 1×1 and 3×3 convolutions to construct an information distillation module, distilling the current feature map through channel separation, achieving real-time performance while maintaining reconstruction accuracy. Based on IDN, IMDN [11] introduces a multi-information distillation module that extracts a part of useful features each time and integrates the remaining features into the distillation step of the next stage. After completion, the features extracted in each step are connected together. Subsequently, RFDN [13] combines feature distillation connections and shallow residual blocks to construct a residual feature distillation block, achieving a better performance than IMDN with fewer parameters.

In pruning methods, SCCVLAB [16] uses a fine-grained channel pruning strategy to address image super-resolution problems, achieving satisfactory results. SMSR [7] prunes redundant computations by learning spatial and channel masks, achieving a better performance with an improved inference efficiency.

Although the aforementioned methods are lightweight and efficient, the quality of SR reconstruction still requires significant improvement.

### 2.2. Attention Mechanism of Image SR

Researchers in the field of image super-resolution have adopted the attention mechanism, which was initially developed for natural language processing tasks [17,18], and has proven effective in image super-resolution.

Hu et al. [18] proposed using channel attention (CA), which assigns a weight to each feature channel based on its significance and improves the feature representation by amplifying the features with high weights and suppressing those with low weights. Hui et al. [15] enhanced the channel attention mechanism with contrast-aware channel attention (CCA), which assigns channel weights according to the sum of the standard deviation and the mean. Wang et al. [19] introduced efficient channel attention (ECA), which uses 1D convolution to efficiently capture dependencies across channels, to make the attention mechanism lighter.These attention mechanisms exhibit state-of-the-art performance in SR tasks [4,8,15].

Some studies have introduced spatial attention to enrich the feature map. Wang et al. [20] proposed an additional attention mechanism, non-local attention, which captures global context information by computing pixel-to-pixel dependencies. Nevertheless, this mechanism incurs a substantial computational overhead. To address this issue, Liu et al. [13] proposed enhanced spatial attention (ESA), which reduces the channel dimensions by employing a 1×1 convolutional layer followed by a stride convolution to expand the receptive field. The max pooling operation with a large window and stride then focuses on the feature’s crucial spatial information. EFDN [10] and BSRN [6] also demonstrate superior performance with ESA.

Guo et al. [21] proposed a novel linear attention mechanism named Large Kernel Attention (LKA) that utilizes the large receptive field of large convolutional kernels to achieve the effects of adaptability and long-range correlations similar to self-attention. The LKA attention mechanism has demonstrated excellent performance in various computer vision tasks [22,23]. However, the use of large convolutional kernels in LKA can introduce a significant computational burden. To address this, we decompose the large convolutional kernels in LKA into smaller ones, achieving results comparable to LKA while significantly reducing the computational requirements.

### 2.3. Factorized Convolution

Factorized convolution has emerged as a promising technique in efficient neural network design. It involves breaking down a standard convolution operation into multiple smaller convolution operations, typically aimed at reducing the computational complexity and model parameters. This technique has found widespread application in various computer vision tasks, including image classification, object detection, and semantic segmentation.

One common form of factorized convolution is depth-wise Separable Convolution, where a standard convolution layer is decomposed into two independent operations: depth-wise convolution and point-wise convolution. Depth-wise convolution independently filters each input channel spatially, while point-wise convolution combines the filtered outputs from each channel. This factorization significantly reduces the number of parameters, resulting in more efficient models.

Recent research has demonstrated the immense potential of factorized convolution in enhancing the efficiency of neural networks. For instance, MobileNet [24] introduced depth-wise Separable Convolution, creating lightweight models suitable for mobile devices. ERFNet [25] factorized 3×3 convolutions into 3×1 and 1×3 convolutions, achieving substantial performance improvements in semantic segmentation tasks. Subsequent studies like DABNet [26], LEDNet [27], and MSCFNet [28] have further improved upon this technique and successfully applied it to their respective tasks, further emphasizing the importance of factorized convolution in efficient network design.

While factorized convolution has been successful in tasks such as image classification and object detection, its application in enhancing the efficiency of super-resolution neural networks has been relatively limited. Despite the successes observed in image classification and object detection tasks, the untapped potential of factorized convolution in improving the efficiency of super-resolution neural networks remains largely unexplored.

To address this gap, we propose an innovative approach in this work, applying factorized convolution to super-resolution networks. Our method fully leverages the advantages of factorized convolution to create highly efficient and lightweight architectures capable of delivering high-quality image super-resolution results.

## 3. Method

### 3.1. Network Structure

**Figure 2:**
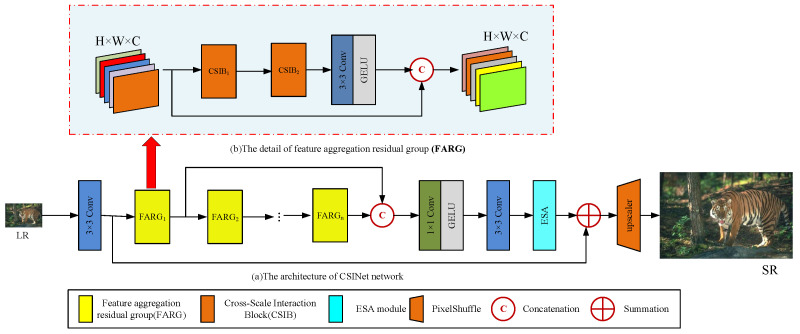
An overview of our CSINet network. (**a**) The architecture of CSINet network, (**b**) the details of the feature aggregation residual group (FARG).

Our CSINet intends to reconstruct HR images by leveraging the work of RFDN [13] and blueprint separable residual network (BSRN) [6]. Figure 2 depicts the architecture of CSINet, which comprises four modules: shallow feature extraction, multiple stacked feature aggregation residual group, dense feature fusion (DFF), and image reconstruction. The goal of the shallow feature extraction module is to extract low-level image features. The multiple stacked feature aggregation residual group is intended to aggregate and refine features from multiple scales. The dense feature fusion (DFF) module combines features from multiple scales, utilizing the attention mechanism to highlight important features and suppress irrelevant ones. The image reconstruction module then reconstructs the HR image based on the fused features.

Shallow Feature Extraction. Given a low-quality input image $I^{\mathrm{LR}}\in R^{H\times W\times C}$, the shallow feature $F_{0}$ is extracted by a 3×3 convolutional layer. This process can be expressed as

(1) $$F_{0}=f_{c3\times 3}\left(I^{\mathrm{LR}}\right)$$

where $f_{cn\times m\_sk}$ denotes an n×m convolutional layer with stride *k* for the shallow feature extraction; this convolution layer provides straightforward mapping from the input image space to a higher-dimensional feature space.

Multiple Stacked Feature Aggregation Residual Group. To extract deep features, we use a non-linear mapping module that consists of several stacked feature aggregation residual groups (FARGs). The output of the *i*-th FARG $F_{i}$ can be expressed as follows:

(2) $$F_{i}={\mathrm{FARG}}_{i}\left(F_{i-1}\right),i=1,2,\cdots ,N$$

where ${\mathrm{FARG}}_{i}\left(\cdot \right)$ is the function of *i*-th FARG and the corresponding output denoted by $F_{i}$. More details of FARG unit will be given in Section 3.3.

Dense Feature Fusion (DFF). To combine hierarchical features from all layers, the outputs of these FARGs are concatenated and sent to a DFF module consisting of a 1×1 convolution, a GELU, and a 3×3 convolution. The feature is then refined using an ESA attention module. This procedure can be described as

(3) $$\begin{array}{cc} F_{\mathrm{DEF}}\; & =\mathrm{Fusion}(\mathrm{Concat}\left(F_{1},F_{2},\cdots ,F_{N}\right))\\ F_{\mathrm{fused}} & =\mathrm{ESA}(F_{\mathrm{DEF}}) \end{array}$$

and

$$\mathrm{Fusion}=f_{c3\times 3}\circ f_{\mathrm{GELU}}\circ f_{c1\times 1}$$

where $\mathrm{Concat}(F_{1},F_{2},\cdots ,F_{N})$ is the concatenation of features generated by all FARG units, $F_{DFF}$ is the aggregated feature, ESA denotes the spatial attention (ESA) (further details will be provided in Section 3.2), $f_{\mathrm{GELU}}\left(\cdot \right)$ is the Gaussian error linear unit activation function, and ∘ denotes function composition.

Image Reconstruction. The image reconstruction module consists of a 3×3 convolutional layer and a Pixel-Shuffle layer. The reconstruction stage is expressed as

(4) $$\begin{array}{cc} F_{\mathrm{comb}} & =F_{\mathrm{fused}}+F_{0}\\ I^{\mathrm{SR}}\;\;\; & =f_{\mathrm{up},\mathrm{ps}}\left(f_{c3\times 3}\left(F_{\mathrm{comb}}\right)\right) \end{array}$$

where $I^{\mathrm{SR}}$ denotes the super-resolution result of the network, $f_{\mathrm{up},\mathrm{ps}}$ indicates the Pixel Shuffle operation.

Loss Function. We utilize the L1 loss to optimize the parameters of our CSINet model as

(5) $$L={∥I^{\mathrm{HR}}-I^{\mathrm{SR}}∥}_{1}$$

where $I^{\mathrm{SR}}$ is the super-resolution result of the network, and $I^{\mathrm{HR}}$ denotes the corresponding high-resolution image.

### 3.2. Attention Modules

#### 3.2.1. Efficient Large Kernel Attention (ELKA)

Guo et al. [21] introduced an innovative linear attention mechanism known as Large Kernel Attention (LKA), which leverages the expansive receptive field provided by large convolution kernels to attain adaptability and long-range correlation effects, akin to self-attention mechanisms. LKA has demonstrated remarkable efficacy, particularly in SR tasks [22,23]. Nonetheless, the utilization of large convolution kernels in LKA imposes a substantial computational burden.

To address this issue, we adopted two pivotal strategies. First, we decomposed the 2D convolution kernel in the deep convolution layer of LKA into a sequence of cascaded horizontal and vertical 1D convolution kernels. Specifically, a K×K spatial convolution was deconstructed into a K×1 depth-wise convolution and a 1×K depth-wise convolution. This decomposition effectively curtails the quadratic increase in the number of parameters in LKA as the convolution kernel size grows, all the while preserving performance quality.

**Figure 3:**
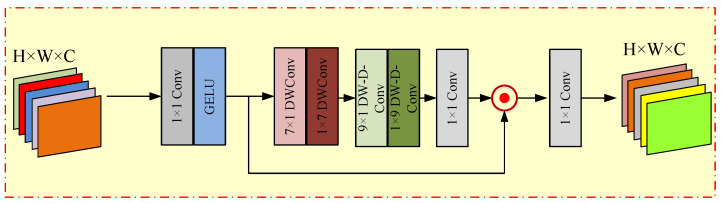
Efficient Large Kernel Attention (ELKA).

Secondly, we introduced a 1×1 convolution layer both preceding and following the depth convolution operation, facilitating information interaction across channels. This groundbreaking module is denoted as ELKA. The overall architecture of ELKA is visually depicted in Figure 3.

The ELKA module consists of three parts: (1) Spatial local convolution: including two cascaded depth-wise convolution operations with convolution kernel sizes of 7 × 1 (DW-Conv7 × 1) and 1 × 7 (DW-Conv1 × 7). (2) Spatial global convolution: Contains two cascaded deep-wise dilated convolution operations with convolution kernel sizes of 9 × 1 (DW-D-Conv9 × 1) and 1 × 9 (DW-D-Conv1 × 9). (3) Two channel convolutions: These two channel convolutions are applied at the beginning and end of the module. The expression of ELKA operation is as follows:

(6) $$\begin{array}{cc} F_{1}^{\mathrm{ELKA}} & =f_{\mathrm{GELU}}\left(f_{c1\times 1}\left(F_{\mathrm{in}}^{\mathrm{ELKA}}\right)\right)\\ F_{2}^{\mathrm{ELKA}} & =f_{\mathrm{dw}1\times 7}\left(f_{\mathrm{dw}7\times 1}\left(F_{1}^{\mathrm{ELKA}}\right)\right)\\ F_{3}^{\mathrm{ELKA}} & =f_{\mathrm{dw}1\times 9\_rd}\left(f_{\mathrm{dw}9\times 1\_rd}\left(F_{2}^{\mathrm{ELKA}}\right)\right)\\ F_{4}^{\mathrm{ELKA}} & =f_{c1\times 1}\left(F_{3}^{\mathrm{ELKA}}\right)\\ F_{\mathrm{out}}^{\mathrm{ELKA}} & =f_{c1\times 1}\left(F_{1}^{\mathrm{ELKA}}⊙F_{4}^{\mathrm{ELKA}}\right) \end{array}$$

where $F_{\mathrm{out}}^{\mathrm{ELKA}}$ denotes the output of the ELKA module; $f_{\mathrm{GELU}}\left(\cdot \right)$ is the gelu activation function; $f_{\mathrm{dw}n\times m}$ indicates the n×m depth-wise convolution operation, $f_{\mathrm{dw}n\times m\_rd}$ is n×m depth-wise dilated convolution operation with dilated rate *d*, ⊙ indicates hadamard product.

Compared to the standard LKA design, ELKA can achieve comparable performance while exhibiting a lower computational complexity and memory.

#### 3.2.2. Enhanced Spatial Attention

**Figure 4:**
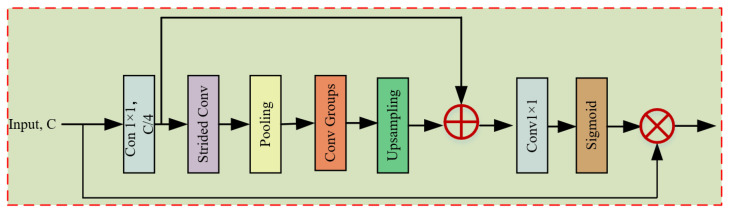
Enhanced Spatial Attention (ESA).

Enhanced Spatial Attention (ESA) is a lightweight and effective spatial attention mechanism [13], as shown in Figure 4. To reduce the computational cost, the ESA module first reduces the number of channels using 1×1 convolution. For enlarging the receptive field further, ESA first halves the size of the feature map using a 1×1 convolution with stride 2, and then adds a 7×7 max pooling layer with stride 3 to reduce the spatial dimension. Following a series of 3×3 convolutions to determine the interdependence of the feature map’s spatial dimensions, a bilinear interpolation is then used to restore the feature map to its original size and then concatenate the features obtained from the previous feature map. Then, a 1×1 convolutional layer is utilized to restore the number of channels of the feature map to its initial value. The attention mask is then generated by the sigmoid activation function and multiplied by the input features to produce an output feature map with long-distance dependence. Given the input characteristics $F_{\mathrm{in}}^{\mathrm{ESA}}$ of ESA, the preceding operations can be described as follows:

(7) $$\begin{array}{cc} F_{1}^{\mathrm{ESA}} & =f_{c1\times 1}\left(F_{\mathrm{in}}^{\mathrm{ESA}}\right)\\ F_{2}^{\mathrm{ESA}} & =\mathrm{Enlarge}(F_{1}^{\mathrm{ESA}})\\ F_{3}^{\mathrm{ESA}} & =F_{1}^{\mathrm{ESA}}+f_{c1\times 1}\left(F_{2}^{\mathrm{ESA}}\right)\\ w & =f_{\mathrm{sigmoid}}\left(f_{\mathrm{up},\mathrm{bi}}\left(F_{3}^{\mathrm{ESA}}\right)\right)\\ F_{\mathrm{out}}^{\mathrm{ESA}} & =F_{\mathrm{in}}^{\mathrm{ESA}}⊗w \end{array}$$

and

(8) $$\mathrm{Enlarge}=f_{\mathrm{upsample}}\circ \underset{\mathrm{conv}\;\mathrm{group}}{\underset{︸}{\left({◯}_{i}f_{i,c3\times 3}\right)}}\circ f_{m7\times 7\_s3}\circ f_{c1\times 1\_s2}$$

where $F_{\mathrm{out}}^{\mathrm{ESA}}$ denotes the output of the ESA module; $f_{mn\times m\_sk}$ represents the n×m max pooling layer with stride *k*; $f_{\mathrm{up},\mathrm{bi}}$ is the bilinear interpolation operation; $f_{\mathrm{upsample}}$ is the upsampling operation; $f_{\mathrm{sigmoid}}\left(\cdot \right)$ is the sigmoid activation function; and ⊗ indicates the element-wise product.

### 3.3. Feature Aggregation Residual Group (FARG)

The feature distillation technique introduced in RFDN [13] has proven effective in reducing the number of parameters while improving performance. Nevertheless, recent studies [5] have indicated that eliminating the feature distillation branch can lead to a reduction in the runtime and computational cost. Motivated by the findings of [29], we have developed the Feature Aggregation Residual Group (FARG) architecture, which is depicted in Figure 2a.

FARG has been designed to be an efficient network module. It comprises two Cross-Scale Interaction Blocks (CSIB), a 3×3 depth-wise convolution layer, and employs the GELU activation function. To begin, FARG processes input features through a pair of CSIBs, a step critical for obtaining deep and robust feature representations. These channel feature enhancement blocks are instrumental in extracting and enriching information from the input features. Next, the channel features undergo convolution through 3×3 convolutional layers, further enhancing the feature representation. This step plays a crucial role in capturing spatial relationships and structural information between the features. Subsequently, the GELU activation function is applied for nonlinear transformation, introducing more complex nonlinear characteristics. This is valuable for enabling the model to better comprehend the intricacies of the data and extract abstract features. Finally, residual operations combine identity mapping with the output features, ensuring that the acquired features effectively integrate with the original input features to better preserve valuable information. This architectural design enhances the network module’s ability to learn and represent complex data features with greater effectiveness. The procedure of FARG can be expressed as

(9) $$\begin{array}{cc} F_{1}^{\mathrm{FARG}} & =\underset{\mathrm{CSIB}\;\mathrm{group}}{\underset{︸}{\left({◯}_{i}{\mathrm{CSIB}}_{i}\right)}}(F_{\mathrm{in}}^{\mathrm{FARG}})\\ F_{\mathrm{out}}^{\mathrm{FARG}} & =f_{c3\times 3}(f_{GELU}\left(F_{1}^{\mathrm{FARG}}\right)+F_{\mathrm{in}}^{\mathrm{FARG}}) \end{array}$$

where $F_{\mathrm{in}}^{\mathrm{FARG}}$ and $F_{\mathrm{out}}^{\mathrm{FARG}}$ are the input and output of the FARG, respectively; CSIB stands for the Cross-Scale Interaction Block, which will be introduced later; ${◯}_{i}{\mathrm{CSIB}}_{i}$ is a CSIB group as a sequence of CSIB blocks; here, two CSIBs are applied for our network; and $f_{\mathrm{GELU}}\left(\cdot \right)$ is the Gaussian error linear unit activation function.

### 3.4. Cross-Scale Interaction Block (CSIB)

**Figure 5:**
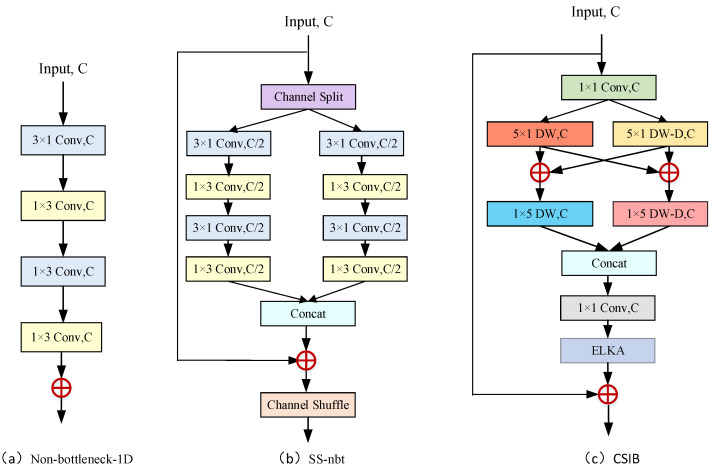
The structures of different residual modules. (**a**) Non-bt-1D [25]. (**b**) SS-nbt [27]. (**c**) Our CSIB. ‘C’ is the number of the input channels, ‘DW’ indicates a depth-wise convolution, and ‘DW-D’ denotes a depth-wise dilated convolution.

To create an efficient architecture, we propose the efficient cross-scale interaction block (CSIB), inspired by the work of Romera [25], Wang [27], Li [26], and Gao [28]. The primary focus of the CSIB’s design is on cross-scale information interaction, taking into consideration the limitations of existing methods in terms of the feature representation capability and efficiency. The CSIB incorporates factorized depth-wise dilated convolutions and residual connections for efficient representation learning, as shown in Figure 5c. In contrast to the single-branch structure of the Non-bottleneck-1D module proposed by Romera [25] and the dual-branch structure of the SS-nbt module proposed by Wang [27], CSIB utilizes an effective cross-scale interaction technique to integrate cross-scale contextual information. This architecture is intended to strike a balance between accuracy and parameters, allowing for improved feature representation and enhanced computational efficiency.

Firstly, CSIB employs a 1×1 convolution layer to decrease the number of parameters and expedite the training process. Following [27,28], we employ a dual-branch structure to simultaneously extract local and multi-scale contextual information. Unlike SS-nbt [27], we replace the factorization convolution with depth-wise factorization convolution to further reduce the parameters in the first branch, which can extract local information. The second branch applies factorization convolution to the depth-wise dilated convolution in order to enlarge the receptive field, thereby capturing global context information. According to previous studies [26,28], dilated convolution may result in gridding artifacts; therefore, we employ depth-wise dilated convolutions with varying dilation rates in various CSIBs. To integrate the cross-scale contextual information of different branches, we perform an element-wise sum of the feature maps extracted by the 5×1 convolutions in the two branches and feed them to a subsequent 1×5 convolution in each branch. In this manner, the extracted feature maps from the two branches can interact.

Considering that concatenation operations are more effective than addition operations, we use concatenation to merge the convolution outputs of two branches. Due to the fact that the receptive fields of the two branches are different sizes, 1×1 convolution is used to promote the fusion of the contextual information extracted by the two branches, strengthen information interaction, and improve feature representation. Following this is an ELKA module for extracting distinguishing characteristics. Finally, the shortcut connection is utilized to preserve the previous functionality, and then we join this into the subsequent CSIB. The operations of CSIB can be expressed as

(10) $$\begin{array}{cc} F_{1}^{\mathrm{CSIB}} & =f_{c1\times 1}\left(F_{\mathrm{in}}^{\mathrm{CSIB}}\right)\\ F_{2}^{\mathrm{CSIB}} & =f_{\mathrm{dw}5\times 1}\left(F_{1}^{\mathrm{CSIB}}\right)+f_{\mathrm{dw}5\times 1\_rd}\left(F_{1}^{\mathrm{CSIB}}\right)\\ F_{3,L}^{\mathrm{CSIB}} & =f_{\mathrm{dw}1\times 5}\left(F_{2}^{\mathrm{CSIB}}\right)\\ F_{3,R}^{\mathrm{CSIB}} & =f_{\mathrm{dw}1\times 5\_rd}\left(F_{2}^{\mathrm{CSIB}}\right)\\ F_{4}^{\mathrm{CSIB}} & =\mathrm{ELKA}(f_{c1\times 1}\left(\mathrm{Concat}\left(F_{3,L}^{\mathrm{CSIB}},F_{3,R}^{\mathrm{CSIB}}\right)\right)\\ F_{\mathrm{out}}^{\mathrm{CSIB}} & =F_{4}^{\mathrm{CSIB}}+F_{\mathrm{in}}^{\mathrm{CSIB}} \end{array}$$

where $f_{\mathrm{dw}n\times m}$ indicates the n×m depth-wise convolution operation, $f_{\mathrm{dw}n\times m\_rd}$ is n×m depth-wise dilated convolution operation with dilated rate *d*, ELKA indicates efficient large kernel attention module, $\mathrm{Concat}(F_{3,L}^{\mathrm{CSIB}},F_{3,R}^{\mathrm{CSIB}})$ is the concatenation of features generated by $F_{3,L}^{\mathrm{CSIB}}$, $F_{3,R}^{\mathrm{CSIB}}$.

## 4. Experiments

### 4.1. Experiment Setup

#### 4.1.1. Datasets and Metrics

Following previous research [5,7,10,11,12,13], we train our models using the recently popularized dataset DIV2K [30] with 800 high-quality images. Five standard benchmark datasets are used to evaluate our models: Set5 [31], Set14 [32], BSD100 [33], Urban100 [34], and Manga109 [35]. To objectively assess the performance of our model, we convert the image to the YCbCr color space and compute the peak signal-to-noise ratio (PSNR) and structural similarity (SSIM) metrics on the luminance channel.

PSNR stands for Peak Signal-to-Noise Ratio and is a measure of image quality that compares the original image to the compressed or distorted image. It is defined as

(11) $$\mathrm{PSNR}=10\log_{10}\left(\frac{MAX_{I}^{2}}{MSE}\right)$$

where $MAX_{I}$ is the maximum pixel value of the image, and MSE is the Mean Squared Error between the original and compressed/distorted images. Higher values of the PSNR indicate a better image quality.

SSIM stands for Structural Similarity Index and is a metric that compares the structural similarity of two images, taking into account the luminance, contrast, and structure. It is defined as:

(12) $$\mathrm{SSIM}\left(x,y\right)=\frac{\left(2\mu_{x}\mu_{y}+C_{1}\right)\left(2\sigma_{xy}+C_{2}\right)}{\left(\mu_{x}^{2}+\mu_{y}^{2}+C_{1}\right)\left(\sigma_{x}^{2}+\sigma_{y}^{2}+C_{2}\right)}$$

where *x* and *y* are the two images being compared, $\mu_{x}$ and $\mu_{y}$ are their respective means, $\sigma_{x}^{2}$ and $\sigma_{y}^{2}$ are their respective variances, and $\sigma_{xy}$ is their covariance. $C_{1}$ and $C_{2}$ are constants used to avoid instability when the means are close to zero. The SSIM value ranges between −1 and 1, where a value of 1 indicates perfect similarity.

#### 4.1.2. Training Details

During the training phase, LR training images are generated by downsampling HR images with scaling factors (×2, ×3, and ×4) using bicubic interpolation in MATLAB R2017a. We apply random horizontal or vertical flips and 90° rotations to the training set. In each mini-batch, inputs consisting of 48×48 LR color patches are selected. The Adan optimizer will be used to train our model with the parameters $\beta_{1}=0.98$, $\beta_{2}=0.92$, $\beta_{3}=0.99$, and with an initial learning rate of $1\times 10^{-3}$. In the training stage, we use L1 to train our network for $1\times 10^{6}$ iterations, and reduce the learning rate by half at $6\times 10^{5}$ and $8\times 10^{5}$ iterations. Subsequently, in the fine-tuning stage, we switch to the L2 to fine-tune our network with a learning rate of $2\times 10^{-5}$, and a total of $1\times 10^{5}$ iterations.

We replaced the 3×3 convolution in the FARG model with a 1×1 convolution, creating a smaller CSINet called CSINet-S. We trained CSINet-S using the DIV2K and Flickr2K datasets. During the training process, the input patch size is set to 64×64 and the mini-batch is set to 64. The Adan optimizer will be used to train our model with the parameters $\beta_{1}=0.98$, $\beta_{2}=0.92$, $\beta_{3}=0.99$, and with an initial learning rate of $1\times 10^{-3}$. We use L1 to train our network for $1\times 10^{6}$ iterations, and reduce the learning rate by half at $6\times 10^{5}$ and $8\times 10^{5}$ iterations. Subsequently, in the fine-tuning stage, we switch to the L2 to fine-tune our network with a learning rate of $2\times 10^{-5}$, and a total of $1\times 10^{5}$ iterations.

The proposed networks are implemented using the PyTorch framework and trained on a single NVIDIA 3090 GPU.

### 4.2. Ablation Study

#### 4.2.1. Effectiveness of Dilation Rate

In deep learning-based image super-resolution methods, the receptive field size of the network is an important factor that affects the ability of the network to capture spatial information from the input image. The dilation rate is a common way to adjust the receptive field size of a CNN. A larger dilation rate means a larger receptive field, which can capture more global contextual information, while a smaller dilation rate means a smaller receptive field, which can capture more local details.

As shown in Table 1, we conducted extensive experiments to investigate the effects of different dilation rates on the image super-resolution performance. Specifically, we adopted the concept from [26,28] and tested seven different dilation configurations. Our experimental results demonstrate that the choice of the dilation rate has a significant impact on the quality of the super-resolved images.

Among the tested dilation configurations, we found that setting the dilation rates to (1,3,3,5) consistently produces superior results across multiple benchmark datasets. These results are in line with previous studies that have also shown the effectiveness of large dilation rates in image super-resolution tasks.

#### 4.2.2. Effectiveness of CSIB

**Figure 6:**
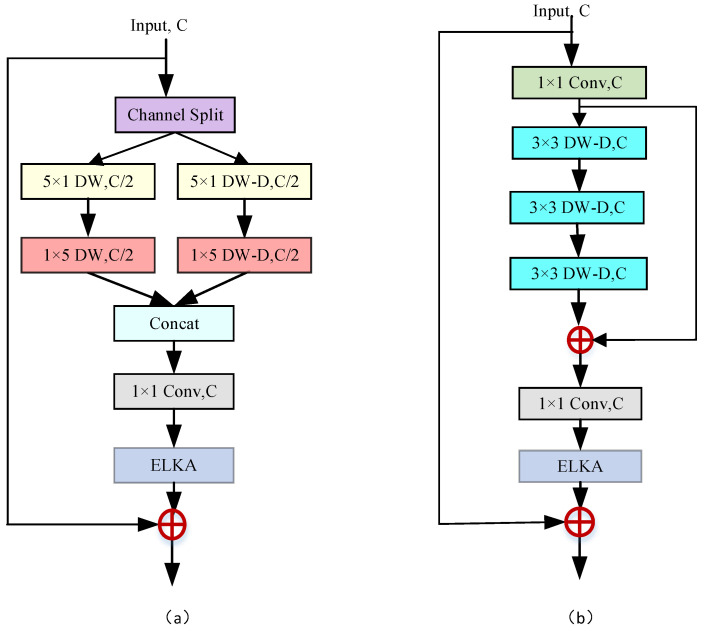
Two different CSIB modules. (**a**) MFFB. (**b**) CDFB. ‘C’ is the number of the input channels, ‘DW’ indicates a depth-wise convolution, and ‘DW-D’ denotes a depth-wise dilated convolution.

The CSIB is intended to enhance the model’s reconstruction performance by effectively fusing multi-scale features from different branches. This is achieved through its parallel branching and cross-fusion structures. To evaluate the effectiveness of the CSIB, two similar modules were designed for comparative analysis (Figure 6).

The Multi-Branch Feature Fusion Block (MFFB), splits the input features into two branches using channel splitting and halving operations. The multi-scale contextual information is then extracted from these two branches using depth-wise factorized convolution. The Cascade Dilated Fusion Block (CDFB), employs a cascade structure of three 3×3 depth-wise dilated convolutions instead of the two-branch structure used in MFFB. Both of these modules were integrated into corresponding SR networks, named a Multi-Branch Feature Fusion Network (MFFNet) and Cascaded Dilated Fusion Network (CDFNet), respectively. Extensive experiments were conducted to evaluate the performance of these three SR networks, with the results shown in Table 2.

In terms of the reconstruction accuracy, the outcomes of these experiments clearly indicate that the CSIB is superior to both MFFB and CDFB. The CSIB achieved greater PSNR and SSIM values, indicating that the reconstructed images were more accurate. In addition, it did so with fewer parameters and at a lower computational cost, proving the efficacy of the interactive fusion structure in the SR reconstruction procedure. Compared to CDFB, CSIB not only requires fewer parameters, but also demonstrates a significant performance advantage in terms of reconstruction. This demonstrates the CSIB’s ability to not only have a large receptive field, but also effectively combine complementary information from multiple scales to improve the model’s representational capabilities.

**Figure 7:**
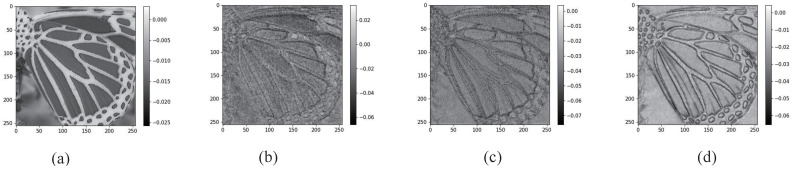
Visualized feature maps processed by different convolution designs. (**a**) Input feature. (**b**) Feature processed by the CDFB. (**c**) Feature processed by the MFFB. (**d**) Output feature of CSIB.

The visual analysis of CDFB, MFFB, and CSIB is presented in Figure 7. As depicted in Figure 7a, CDFB shows promising results in recovering a portion of the butterfly’s streak profile, albeit with some blurring. In contrast, Figure 7b presents MFFB which stands out due to its ability to extract more details of the stripes. This enhanced performance is attributed to its effective use of multi-scale feature extraction modules, which facilitates the recovery of intricate details with remarkable precision.

Furthermore, the proposed CSIB, shown in Figure 7c, also utilizes multi-scale feature extraction modules, leading to a superior restoration performance when compared to the aforementioned models. CSIB excels in reconstructing high-frequency details and edge information with exceptional clarity, as evidenced in the results. The findings highlight the proficiency of CSIB in structural texture restoration and demonstrate the immense potential of deep learning models in image processing applications.

#### 4.2.3. Effectiveness of Factorized Convolution

To validate the effectiveness of factorized convolution, we replaced it with regular convolution in CSIB, denoted as “w/RC”.

Upon reviewing the results in Table 3, it is clear that the inclusion of factorized convolution leads to a reduction of 14 K parameters and a decrease of 0.8 G FLOPs compared to regular convolution. Simultaneously, PSNR and SSIM exhibit improvements across all benchmark datasets. Furthermore, the inference time decreased by 1.24 ms. These findings indicate that the introduction of factorized convolution not only enhances the model’s lightweight characteristics but also contributes to significant performance improvements.

#### 4.2.4. Effectiveness of ELKA and ESA

The ablation studies on the two attention modules—ELKA, and ESA—are presented in Table 4. The results indicate that ELKA is a highly effective module. We observed a significant decrease in the network performance when ELKA was removed, with a decrease of approximately 0.2 dB in the Set5 and Set14 datasets, and a decrease of over 0.4 dB in the Urban100 and Manga109 datasets. Furthermore, ESA has a positive impact on the model’s performance, as evidenced by a substantial decmidrule in the performance when ESA is removed.

These findings demonstrate that combining ELKA and ESA can effectively increase the model’s capacity. It is noteworthy that ELKA provides a more computationally efficient way to incorporate global information, while ESA modules can enhance the local feature representation. Thus, the combination of these attention modules offers a well-balanced and effective solution to improve the model’s performance.

**Figure 8:**
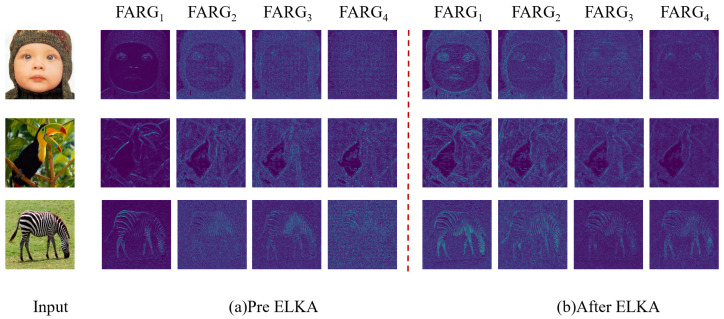
Visualized feature maps of the four FARGs. (**a**) Visualization feature maps of the four FARGs before ELKA. (**b**) Visualization feature maps of the four FARGs after ELKA. The values are calculated by averaging the feature maps and normalized in range 0 to 1.

To further observe the benefits produced by our ELKA module, we visualize the feature maps before and after ELKA for different FARGs, as shown in Figure 8. It can be observed that the ELKA module enhances high-frequency information, making the edges and structural textures in the output features clearer.

### 4.3. Comparison with the SOTA SR Methods

To verify the effectiveness of the proposed model, we compare our CSINet model with 14 lightweight state-of-the-art SISR methods, including SRCNN [1], VDSR [2], CARN [14], IDN [15], MAFFSRN [36], SMMR [7], IMDN [11], PAN [8], LAPAR-A [12], RFDN [13], Cross-SRN [37], FDIWN [38], RLFN [5], and BSRN [6]. The results of the comparisons are presented in Table 5. To assess the model’s size, we used two metrics: the number of parameters and the number of operations (Multi-Adds), calculated on a high-resolution image of 1280×720. Our method achieved outstanding results on all the datasets with various scaling factors, outperforming most of the other state-of-the-art networks in both the PSNR and SSIM measurements. Despite having fewer parameters and Multi-Adds, our CSINet outperformed techniques such as LAPAR-A, RFDN, Cross-SRN, and even the RLFN, which was awarded second place in the sub-track2 (Overall Performance Track) of the NTIRE 2022 efficient super-resolution challenge. These results illustrate the effective balance between image quality and computational efficiency that our method achieves.

We have incorporated the Non-Reference Image Quality Evaluator (NIQE) into our evaluation metrics to provide a more comprehensive analysis of the performance of our model compared to other lightweight models, including VDSR, CARN, IMDN, PAN, EFDN, and RLFN, as shown in Table 6. In the comparison, we computed the NIQE scores for the outputs of our model and the aforementioned lightweight models. The NIQE score measures the naturalness of an image, with lower scores indicating a better image quality. Our model achieved comparable or slightly lower NIQE scores compared to these models, indicating that our model produces images with similar or slightly better naturalness. These results suggest that our model not only performs competitively in terms of traditional evaluation metrics such as PSNR and SSIM but also maintains or enhances the perceptual quality of the super-resolved images according to the NIQE score. It demonstrates the effectiveness of our lightweight model in preserving image quality while reducing the computational complexity.

**Figure 9:**
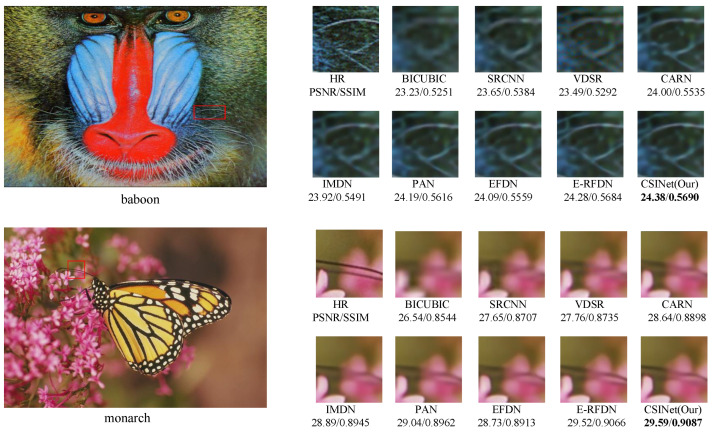
Visual comparison of the Set 14 dataset for ×4 SR.

**Figure 10:**
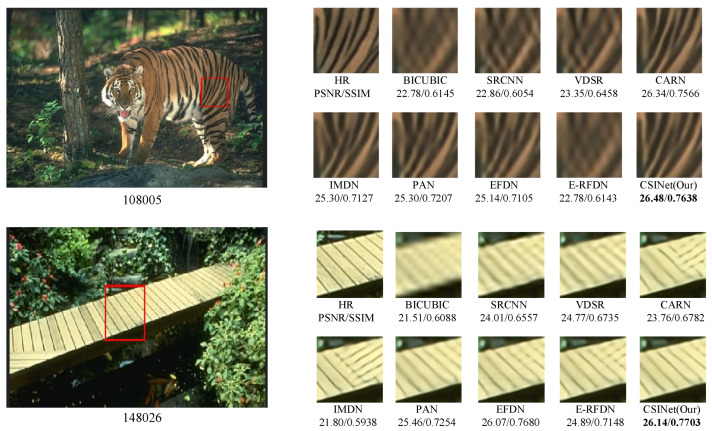
Visual comparison on the BSD100 dataset for ×4 SR.

**Figure 11:**
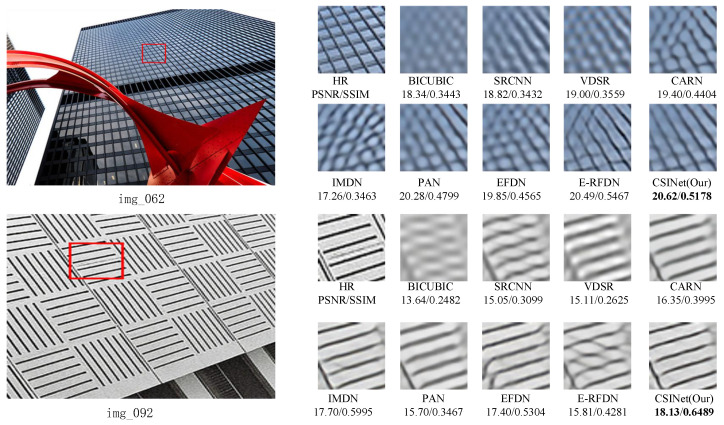
Visual comparison on the Urban100 dataset for ×4 SR.

The visual comparisons of our method with several state-of-the-art methods are presented in Figure 9, Figure 10 and Figure 11. The results demonstrate the superiority of our method in terms of the image quality.

For Set14, we compared the models’ ability to reconstruct the “baboon” and “monarch” images. Our findings suggest that while the SRCNN [1] and VDSR [2] models recovered most of the stripe contours, their reconstructions still exhibited blurriness. In contrast, our proposed model, CSINet, was able to reconstruct high-frequency details with greater clarity. For the “monarch” image, CSINet was also superior in reproducing the butterfly antennae with greater clarity.

On the BSD100 dataset, we evaluated the performance of the models on the “108005” and “148026” images. Our results indicate that Bicubic failed to reproduce the basic texture features when reconstructing the details of the stripes on the tiger. While other models, such as CARN [14], IMDN [11], PAN [8], and EFDN [10], could recover more stripe details, their reconstructed images still exhibited some blurriness. In contrast, CSINet was able to reconstruct high-frequency details with greater clarity, outperforming all the other models. For the “148026” image, CSINet also produced reconstructed images with clear texture and rich details, which were closer to the real images than the other models.

Finally, on the Urban100 dataset, we evaluated the models’ ability to restore the “img_092” image. Our results suggest that most of the models, except for Bicubic, could restore the horizontal stripes of the building facade but still exhibited some blurriness. In contrast, the reconstructed images from CSINet had clear texture and rich details, approaching perfection. Similarly, for the “img_062” image in the Urban100 test set, the reconstructed images using Bicubic, SRCNN [1], and VDSR [2] were severely distorted and blurry. While the reconstructed results using CARN [14], IMDN [11], PAN [8], EFDN [10], and E-RFDN [13] were slightly clearer, the glass window grids were distorted and deformed. In contrast, the reconstructed images using CSINet proposed in this study had clear texture and rich details, which were closer to the real images.

Overall, our subjective visual effect comparisons demonstrate that CSINet outperforms other state-of-the-art super-resolution models, providing high-frequency details that are clearer and closer to the real images.

### 4.4. Complexity Analysis

**Figure 12:**
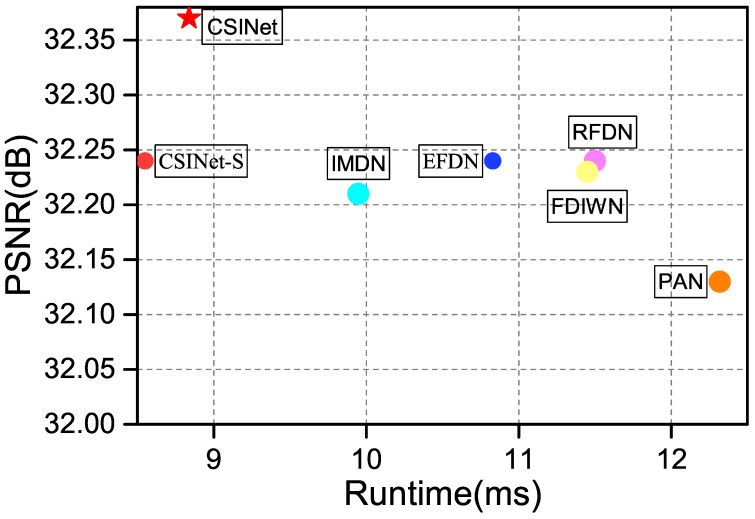
Average running time on Set5 dataset for ×4 SR.

The runtime of a network is a crucial metric, even for lightweight SR algorithms. We conducted comparative experiments on the Set5 dataset (×4) to assess the reconstruction speeds of mainstream networks. The experiments were run on an NVIDIA 3090 GPU with 24 GB RAM. The test images had a spatial resolution of 64×64 pixels. After 10 repeated runs, the average inference times were obtained and are presented in Figure 12. It can be observed that our CSINet not only achieves the fastest reconstruction speed but also delivers the best reconstruction quality, demonstrating the significant advantages of our lightweight CSINet.

**Figure 13:**
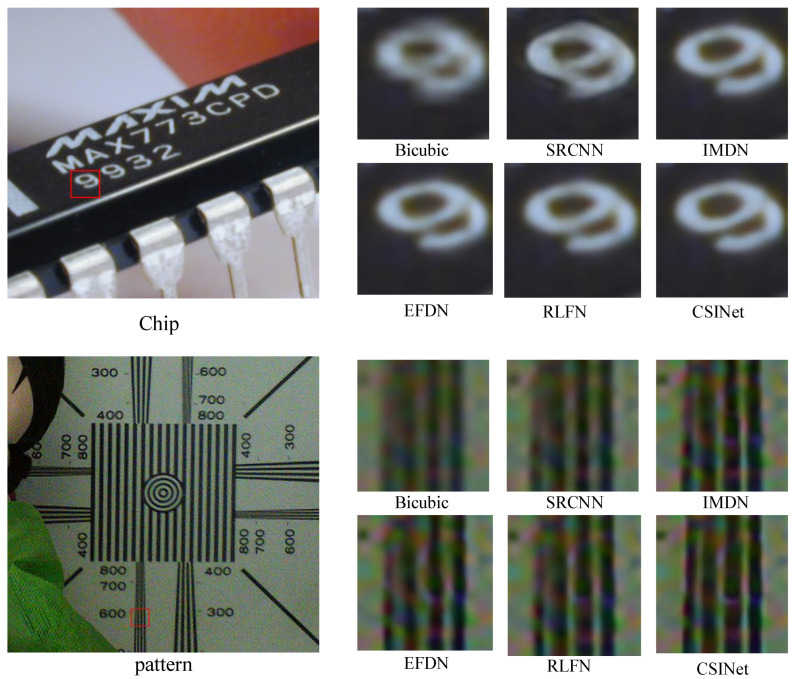
Comparison of super-resolution results on real-world photos; CSINet outperforms state-of-the-art methods with embedded MEC system.

To further validate the lightweight nature of CSINet, we deployed it on the NVIDIA Jetson Xavier NX Developer Kit, known as one of the world’s smallest AI supercomputers for embedded MEC systems. We conducted experiments on real-world photos to evaluate the effectiveness of CSINet in the embedded MEC system. In these scenarios, ground-truth images and downsampling kernels were unavailable. As depicted in Figure 13, our method successfully reconstructs sharper and more accurate images compared to state-of-the-art approaches. This indicates that our lightweight model excels in achieving exceptional super-resolution performance, making it highly suitable for deployment in embedded MEC systems.

### 4.5. Discussions

The effectiveness of the proposed Cross-Scale Interaction Block (CSIB) is a key highlight of our study. CSIB stands out as a crucial component in enhancing the overall performance of CSINet.

Firstly, CSIB is meticulously designed for super-resolution (SISR), integrating cross-scale contextual information using depth-wise convolution and dilated convolution. This design choice proves effective in capturing and leveraging contextual details across different scales, contributing to improved image reconstruction.

Secondly, the incorporation of Efficient Lightweight Kernel Aggregation (ELKA) within CSIB further enhances the model’s representational capacity. ELKA plays a pivotal role in aggregating relevant features efficiently, contributing to the model’s ability to capture intricate details and patterns.

The experimental results underscore the effectiveness of CSIB. In comparison to scenarios where regular convolution is used, the inclusion of factorized convolution within CSIB leads to significant reductions in parameters and FLOPs while simultaneously improving PSNR, SSIM, and reducing the inference time. This indicates that CSIB not only reduces the model complexity but also positively impacts the image quality and computational efficiency.

In visual comparisons with state-of-the-art methods, CSINet equipped with CSIB excels in reconstructing high-frequency details with exceptional clarity. This suggests that the designed cross-scale interaction mechanism within CSIB plays a pivotal role in capturing and utilizing contextual information effectively, resulting in superior image reconstruction.

CSIB emerges as a crucial element contributing to the effectiveness of CSINet. Its innovative design and integration within the network significantly improve image quality, demonstrating the efficacy of the proposed cross-scale interaction strategy in the context of lightweight super-resolution.

## 5. Conclusions

In this paper, we introduce the Cross-Scale Interaction Network (CSINet), a novel architecture designed for lightweight image super-resolution (SISR). Specifically, we present a lightweight Cross-Scale Interaction Block (CSIB) tailored for SISR. This block is carefully crafted to integrate cross-scale contextual information using depth-wise convolution and dilated convolution, leading to an effective reduction in the model complexity. Additionally, the integration of Efficient Lightweight Kernel Aggregation (ELKA) enhances the model’s representational capacity. The proposed network is characterized by its lightweight nature, with only 366K parameters. Extensive experiments conducted on benchmark datasets validate that CSINet outperforms the majority of state-of-the-art lightweight SR methods. Remarkably, it achieves superior results with fewer parameters and Multi-Adds, underscoring its efficiency and effectiveness.

In future work, to enhance the applicability of CSINet in real-time scenarios, optimizing model parameters and the inference time will become crucial for achieving a more lightweight model. This optimization will remain a central focus in our ongoing research, with the goal of ensuring the seamless integration of CSINet into real-time application environments.

## Figures and Tables

**Table 1:** Investigation of different dilate rates. ‘R’ denotes the dilation rates of each depth-wise convolution. These results were recorded after $1\times 10^{6}$ iterations without pre-training and fine-tuning. The best are color red.

Dilation Rate	Set5	Set14	BSD100	Urban100	Manga109
	PNSR/SSIM	PNSR/SSIM	PNSR/SSIM	PNSR/SSIM	PNSR/SSIM
R = (1,2,2,4)	32.26/0.8963	28.68/**0.7845**	27.64/0.7404	26.22/0.7916	30.58/0.9102
R = (1,2,2,6)	32.26/0.8961	28.66/0.7840	**27.65**/0.7403	**26.23**/0.7915	**30.59**/0.9100
R = (1,2,4,6)	32.29/0.8961	**28.69**/0.7843	27.64/0.7404	26.22/0.7916	30.55/0.9099
R = (1,3,5,7)	32.32/**0.8965**	28.67/0.7841	27.63/0.7403	26.21/0.7916	30.56/0.9099
R = (1,3,5,5)	32.29/0.8963	**28.69**/0.7844	27.64/0.7402	26.18/0.7900	30.58/0.9101
R = (1,3,3,5)	**32.34**/**0.8965**	28.68/**0.7845**	27.64/**0.7405**	**26.23**/**0.7918**	30.58/**0.9103**

**Table 2:** Quantitative comparison of three distinct approaches to ×4 SR: MFFNet, CDFNet, and the proposed CSINet. These results were recorded after $1\times 10^{6}$ iterations without pre-training and fine-tuning. The best results are color red.

Method	Params	Multi-Adds	Set5	Set14	BSD100	Urban100	Manga109
			PNSR/SSIM	PNSR/SSIM	PNSR/SSIM	PNSR/SSIM	PNSR/SSIM
MFFNet	311 K	17.3 G	32.17/0.8948	28.55/0.7812	27.53/0.7366	26.01/0.7834	30.32/0.9068
CDFNet	343 K	20.5 G	32.15/0.8944	28.62/0.7827	27.60/0.7389	26.02/0.7859	30.32/0.9075
CSINet	366 K	20.5 G	**32.34**/**0.8965**	**28.68**/**0.7845**	**27.64**/**0.7405**	**26.23**/**0.7918**	**30.58**/**0.9103**

**Table 3:** The results pertaining to the inclusion of factorized convolution in CSIB are presented. “w/RC” denotes the scenario where factorized convolution is replaced with regular convolution, while “w/FC” signifies our model using factorized convolution. The inference time is calculated on Set5 with a scaling factor of ×4. The experiments were executed using an NVIDIA 3090 GPU. The best results are color red.

Method	Params	Multi-Adds	Ave. Time	Set5	Set14	BSD100	Urban100	Manga109
				PNSR/SSIM	PNSR/SSIM	PNSR/SSIM	PNSR/SSIM	PNSR/SSIM
w/RC	380 K	21.3 G	10.08 ms	32.27/0.8961	28.66/0.7837	27.63/0.7398	26.15/0.7886	**30.60**/0.9098
w/FC	366 K	20.5 G	8.84 ms	**32.34**/**0.8965**	**28.68**/**0.7845**	**27.64**/**0.7405**	**26.23**/**0.7918**	30.58/**0.9103**

**Table 4:** Comparison of the number of parameters, Multi-Adds, and mean values of PSNR obtained without ELKA and without ESA and our CSINet on five datasets for ×4 SR. These results were recorded after $1\times 10^{6}$ iterations without pre-training and fine-tuning.The best results are color red.

Method	Params	Multi-Adds	Set5	Set14	BSD100	Urban100	Manga109
			PNSR/SSIM	PNSR/SSIM	PNSR/SSIM	PNSR/SSIM	PNSR/SSIM
w/o ELKA	273 K	15.3 G	32.01/0.8927	28.47/0.7796	27.51/0.7768	25.81/0.7768	30.05/0.9030
w/o ESA	343 K	20.4 G	32.26/0.8960	28.65/0.7840	27.63/0.7401	**26.23**/0.7914	30.55/0.9101
CSINet	366 K	20.5 G	**32.34**/**0.8965**	**28.68**/**0.7845**	**27.64**/**0.7405**	**26.23**/**0.7918**	**30.58**/**0.9103**

**Table 5:** Quantitative comparisons of state-of-the art SR algorithm on five datasets. The best and the second best results are color red and blue, respectively. “Multi-Adds” are computed with a 720p HR image.

Methods	Scale	Params	Multi-Adds	Set5	Set14	BSD100	Urban100	Manga109
				PNSR/SSIM	PNSR/SSIM	PNSR/SSIM	PNSR/SSIM	PNSR/SSIM
Bicubic	×2	-	-	33.66/0.9299	30.24/0.8688	29.56/0.8431	26.88/0.8403	30.80/0.9339
SRCNN [1]	×2	8 K	52.7 G	36.66/0.9542	32.42/0.9063	31.36/0.8879	29.50/0.8946	35.60/0.9663
VDSR [2]	×2	666 K	612.6 G	37.53/0.9587	33.03/0.9124	31.90/0.8960	30.76/0.9140	37.22/0.9750
CARN [14]	×2	1592 K	222.8 G	37.76/0.9590	33.52/0.9166	32.09/0.8978	31.92/0.9256	38.36/0.9765
IDN [15]	×2	553 K	124.6 G	37.83/0.9600	33.30/0.9148	32.08/0.8985	31.27/0.9196	38.01/0.9749
MAFSSRN [36]	×2	402 K	77.2 G	37.97/0.9603	33.49/0.9170	32.14/0.8994	31.96/0.9268	-
SMMR [7]	×2	985 K	131.6 G	38.00/0.9601	33.64/0.9179	32.17/0.8990	32.19/0.9284	38.76/0.9771
IMDN [11]	×2	694 K	158.8 G	38.00/0.9605	33.63/0.9177	32.19/0.8996	32.17/0.9283	38.88/0.9774
PAN [8]	×2	261 K	70.5 G	38.00/0.9605	33.59/0.9181	32.18/0.8997	32.01/0.9273	38.70/0.9773
LAPAR-A [12]	×2	548 K	171.0 G	38.01/0.9605	33.62/0.9183	32.19/0.8999	32.10/0.9283	38.67/0.9772
RFDN [13]	×2	534 K	95 G	38.05/0.9606	33.68/0.9184	32.16/0.8994	32.12/0.9278	38.88/0.9773
Cross-SRN [37]	×2	-	-	38.03/0.9606	33.62/0.9180	32.19/0.8997	32.28/0.9290	38.75/0.92773
FDIWN-M [38]	×2	-	-	-	-	-	-	-
RFLN [5]	×2	527 K	-	38.07/0.9607	33.72/0.9187	32.22/0.9000	32.33/0.9299	-
BSRN [6]	×2	332 K	73.0 G	**38.10**/**0.9610**	33.74/0.9193	32.24/0.9006	32.34/0.9303	**39.14**/**0.9782**
CSINet-S (ours)	×2	248 K	54.6 G	38.06/0.9608	**33.82**/0.9200	32.26/**0.9009**	32.40/0.9313	39.08/0.9780
CSINet (ours)	×2	348 K	77.7 G	38.08/0.9608	33.77/**0.9205**	**32.27**/**0.9009**	**32.45**/**0.9318**	39.00/0.9779
Bicubic	×3	-	-	30.39/0.8682	27.55/0.7742	27.21/0.7385	24.46/0.7349	26.95/0.8556
SRCNN [1]	×3	8 K	52.7 G	32.75/0.9090	29.30/0.8215	28.41/0.7863	26.24/0.7989	30.48/0.9117
VDSR [2]	×3	666 K	612.6 G	33.66/0.9213	29.77/0.8314	28.82/0.7976	27.14/0.8279	32.01/0.9340
CARN [14]	×3	1592 K	118.8 G	34.29/0.9255	30.29/0.8407	29.06/0.8034	28.06/0.8493	33.50/0.9440
IDN [15]	×3	553 K	57.0 G	34.11/0.9253	29.99/0.8354	28.95/0.8013	27.42/0.8359	32.71/0.9381
MAFSSRN [36]	×3	418 K	34.2 G	34.32/0.9269	30.35/0.8429	29.09/0.8052	28.13/0.8521	-
SMMR [7]	×3	993 K	67.8 G	34.40/0.9270	30.33/0.8412	29.10/0.8050	28.25/0.8536	33.68/0.9445
IMDN [11]	×3	703 K	71.5 G	34.36/0.9270	30.32/0.8417	29.09/0.8046	28.17/0.8519	33.61/0.9445
PAN [8]	×3	261 K	39 G	34.40/0.9271	30.36/0.8423	29.11/0.8050	28.11/0.8511	33.61/0.9448
LAPAR-A [12]	×3	544 K	114 G	34.36/0.9267	30.34/0.8421	29.11/0.8054	28.15/0.8523	33.51/0.9441
RFDN [13]	×3	541 K	42.2 G	34.41/0.9273	30.34/0.8420	29.09/0.8050	28.21/0.8525	33.67/0.9449
Cross-SRN [37]	×3	-	-	32.43/0.9275	30.33/0.8417	29.09/0.8050	28.23/0.8535	33.65/0.9448
FDIWN-M [38]	×3	446 K	35.9 G	34.46/0.9274	30.35/0.8423	29.10/0.8051	28.16/0.8528	-
RFLN [5]	×3	-	-	-	-	-	-	-
BSRN [6]	×3	340 K	33.3 G	32.46/0.9277	30.47/0.8449	29.18/0.8068	28.39/0.8567	**34.05**/**0.9471**
CSINet-S (ours)	×3	255 K	25.1 G	34.47/0.9275	30.46/0.8449	29.18/0.8076	**28.37**/0.8573	33.91/0.9464
CSINet (ours)	×3	356 K	35.3 G	**34.49**/**0.9279**	**30.49**/**0.8453**	**29.19**/**0.8077**	**28.40**/**0.8577**	33.93/0.9464
Bicubic	×4	-	-	28.42/0.8104	26.00/0.7027	25.96/0.6675	23.14/0.6577	24.89/0.7866
SRCNN [1]	×4	57 K	52.7 G	30.48/0.8626	27.50/0.7513	26.90/0.7101	24.52/0.7221	27.58/0.8555
VDSR [2]	×4	666 K	612.6 G	31.35/0.8838	28.01/0.7674	27.29/0.7251	25.18/0.7524	28.83/0.8770
CARN [14]	×4	1592 K	90.9 G	32.13/0.8937	28.60/0.7806	27.58/0.7349	26.07/0.7837	30.47/0.9084
IDN [15]	×4	553 K	32.3 G	31.82/0.8903	28.25/0.7730	27.41/0.7297	25.41/0.7632	29.41/0.8942
MAFSSRN [36]	×4	441 K	19.3 G	32.18/0.8948	28.58/0.7812	27.57/0.7361	26.04/0.7848	-
SMMR [7]	×4	1006 K	41.6 G	32.12/0.8932	28.55/0.7808	27.55/0.7351	26.11/0.7868	30.54/0.9085
IMDN [11]	×4	715 K	40.9 G	32.21/0.8948	28.58/0.7811	27.56/0.7353	26.04/0.7838	30.45/0.9075
PAN [8]	×4	272 K	28.2 G	32.13/0.8948	28.61/0.7822	27.59/0.7363	26.11/0.7854	30.51/0.9095
LAPAR-A [12]	×4	548 K	94 G	32.15/0.8944	28.61/0.7818	27.61/0.7366	26.14/0.7871	30.42/0.9074
RFDN [13]	×4	550 K	23.9 G	32.24/0.8952	28.61/0.7819	27.57/0.7360	26.11/0.7858	30.58/0.9089
Cross-SRN [37]	×4	-	-	32.24/0.8954	28.59/0.7817	27.58/0.7364	26.17/0.7881	30.53/0.9088
FDIWN-M [38]	×4	454 K	19.6 G	32.17/0.8941	28.55/0.7806	27.58/0.7364	26.02/0.7844	-
RFLN [5]	×4	543 K	-	32.24/0.8952	28.62/0.7813	27.60/0.7364	26.17/0.7877	-
BSRN [6]	×4	352 K	19.4 G	32.35/0.8962	28.73/0.7847	27.65/0.7387	26.27/0.7908	30.84/**0.9123**
CSINet-S (ours)	×4	266 K	14.7 G	32.24/0.8959	28.72/0.7839	27.64/0.7385	26.22/0.7901	30.68/0.9097
CSINet (ours)	×4	366 K	20.5 G	**32.37**/**0.8971**	**28.78**/**0.7857**	**27.69**/**0.7398**	**26.35**/**0.7932**	**30.85**/0.9117

**Table 6:** The average NIQE for ×4 SR. Red indicates the best performance. The best results are color red.

Method	Scale	Set5	Set14	BSD100	Urban100
VDSR [2]	×4	8.5458	6.9062	6.9890	6.2648
CARN [14]	×4	7.1466	6.2880	6.5794	5.7105
IMDN [11]	×4	6.8819	6.2901	6.5413	5.6889
EFDN [10]	×4	7.074	6.1642	6.5460	**5.6847**
RFLN [5]	×4	7.2805	6.2011	6.5755	5.7244
CSINet	×4	**6.7845**	**6.1613**	**6.5193**	5.7888

## Data Availability

The available online experimental datasets in this paper are https://paperswithcode.com./ (accessed on 1 April 2023).
